# Intermediate Tyrosyl Radical and Amyloid Structure in Peroxide-Activated Cytoglobin

**DOI:** 10.1371/journal.pone.0136554

**Published:** 2015-08-27

**Authors:** Juliana C. Ferreira, Marcelo F. Marcondes, Marcelo Y. Icimoto, Thyago H. S. Cardoso, Aryane Tofanello, Felipe S. Pessoto, Erica G. A. Miranda, Tatiana Prieto, Otaciro R. Nascimento, Vitor Oliveira, Iseli L. Nantes

**Affiliations:** 1 Departamento de Bioquímica, Universidade Federal de São Paulo, São Paulo, SP, Brazil; 2 Laboratório de Nanoestruturas para Biologia e Materiais Avançados, Centro de Ciências Naturais e Humanas, Universidade Federal do ABC, Santo André, SP, Brazil; 3 Grupo de Biofísica Molecular “Sérgio Mascarenhas,” Instituto de Física de São Carlos, Universidade de São Paulo, São Carlos, SP, Brazil; Albany Medical College, UNITED STATES

## Abstract

We characterized the peroxidase mechanism of recombinant rat brain cytoglobin (Cygb) challenged by hydrogen peroxide, tert-butylhydroperoxide and by cumene hydroperoxide. The peroxidase mechanism of Cygb is similar to that of myoglobin. Cygb challenged by hydrogen peroxide is converted to a Fe^4+^ oxoferryl π cation, which is converted to Fe^4+^ oxoferryl and tyrosyl radical detected by direct continuous wave-electron paramagnetic resonance and by 3,5-dibromo-4-nitrosobenzene sulfonate spin trapping. When organic peroxides are used as substrates at initial reaction times, and given an excess of peroxide present, the EPR signals of the corresponding peroxyl radicals precede those of the direct tyrosyl radical. This result is consistent with the use of peroxide as a reducing agent for the recycling of Cygb high-valence species. Furthermore, we found that the Cygb oxidation by peroxides leads to the formation of amyloid fibrils. This result suggests that Cygb possibly participates in the development of degenerative diseases; our findings also support the possible biological role of Cygb related to peroxidase activity.

## Introduction

Globins are heme proteins that are widely found in bacteria, fungi, plants and animals. In vertebrates, four types of globins have been identified: hemoglobin (Hb), myoglobin (Mb), neuroglobin (Ngb) and Cytoglobin (Cygb) [1–3]. Even though the globins have peculiar structural differences, all are characterized by a high α-helice globular structure folded onto the prostetic heme group [1–10]. Hb and Mb have been extensively studied over several decades and are well characterized in terms of their structure, function and evolution [4–10]. However, for Ngb and Cygb, which were discovered more recently, their biological function is an ongoing topic of discussion. A variety of biological roles have been assigned to Cygb, the subject of the present study. These roles include participation in oxygen supply for cells [5,11,12], acting as a reservoir and sensor of molecular oxygen for tissues [13–16], participating in the metabolism of NO^•^ [17–21], stimulating collagen synthesis in processes of tissue regeneration [22], impairing fibrosis of hepatic stellate cells [23,24], modulating the myogenic progenitor cell viability and muscle regeneration [25], protecting cells from oxidative-related damage during ischemic reperfusion injury subsequent to hypoxia [26], participating in some types of cancer, normally associated with its downregulation [27,28]. Cygb is of particular interest in the present study, and we study the participation of Cygb in the oxidative stress of cells. Several pieces of evidences have demonstrated that Cygb is closely related to the production of hydrogen peroxide in cells [25,26]. Endogenous expression of Cygb is upregulated via the exposure of cells to oxidant conditions [25]. However, in cultured N2a neuroblastoma cells, among various stress factors such as hypoxia (5%), calcium, kainic acid, heat shock or high osmolarity, only hydrogen peroxide increased Cygb levels [29]. Similarly, the upregulation of Cygb was also observed in MCF7 breast cancer cell lines challenged by hydrogen peroxide [30]. Although the peroxidase activity of Cygb unequivocally contributes to the decrease of hydrogen peroxide in cells, a real contribution to impair oxidative stress is dependent of the mechanism of peroxide cleavage.

The primary structure of Cygb has 25% identity with Mb [31]. Furthermore, Cygb exhibits the same overall tertiary structure than Mb, namely the globin fold [3,32]. In the absence of an external ligand, Cygb heme iron in ferric and ferrous forms is hexacoordinated and the fifth and sixth ligands are the imidazole rings of HisF8 and HisE7 [13,33–37]. This arrangement is similar to Ngb and to non-symbiotic plant globins, but distinguishes Cygb from pentacoordinated Hb and Mb. Cygb exists in the monomeric or disulfide-linked dimeric forms with repercussions on its spin state and ligand binding. [38, 39]. The demonstration that Cygb can bind molecular oxygen suggests that despite its hexacoordenated structure, the sixth axial ligand can be displaced [40,41]. In this regard, the peroxidase activity of Cygb has been demonstrated in hepatic stellate cells, in vitro with linoleate [42]. Another study demonstrated that the peroxidase activity of Cygb is modulated by the distal histidine 81 [43]. In stellate cells, the peroxidase activity of Cygb had been postulated to protect cells against transformation in fibroblasts in the development of hepatic cirrhosis. However, the demonstration that Cygb reacts with linoleate and generates oxidized products of the lipid raises the possibility that the peroxidase activity of Cygb is not only associated with cell protection [42]. On the other hand, the low concentrations of the enzyme in cells point to a more probable signaling activity. Therefore, the reaction of low amounts of Cygb with lipid peroxides could produce low concentrations of free radicals that should activate the antioxidant defenses of cells. In the present study, we characterized the reactivity of monomeric Cygb with three different peroxides: hydrogen peroxide, tert-butylhydroperoxide (*t*-BuOOH) and cumene hydroperoxide (CumOOH). Cygb was able to react with all of the investigated peroxides and the reactions were accompanied by the formation of free radicals.

## Materials and Methods

### Chemicals

Terrific broth, Isopropyl β-D-thiogalactoside, sodium phosphate, hydrogen peroxide, thioflavin-T, *t*-BuOOH and CumOOH were obtained from Sigma Chemical (St. Louis, MO, USA).

### Cygb Cloning and Expression

Cygb ORF were amplified from total rat brain cDNA (cDNA kit, Invitrogen, NY, USA) using specific oligonucleotide primers designed according to accession number AJ315163 (mCygbF 5'-*CATATG*ATGGAGAAAGTG CCGGGCGACATGGAGATA-3'; mCygbR 5'-*CTCG AG*TGGCCCTGAAGAGGGCAGTGTGGCTGGTAG-3'). The PCR products were cloned into pTZ57R/T vector (Invitrogen) and subcloned into pET26b (+) vector (Novagen, Madison, Wi, USA) using the restriction enzymes *XhoI* and *NdeI* (Fermentas, NY, USANJ). DNA sequencing was confirmed using DyeTerminator chemistry (Invitrogen) on Megabace sequencer hardware (GE, NJ, USA). The cloning strategy generates constructs for recombinant Cygb containing an additional 8 residues (LEHHHHHH) at the C-terminus. Rat Cygb was expressed in *E*.*coli* strain BL21 plysS (Invitrogen) in 1-liter culture flasks using Terrific Broth medium (Sigma) at 20°C for a period of 48 h after induction with 250 μmol.L^-1^ isopropyl β-D-1-thiogalactopyranoside (IPTG). These cultures were harvested by centrifugation, and the cells were lysed using a BugBuster Master Mix system (Novagen) treatment followed by 45 sec, 4 steps of 60 Hz sonication.

For the extraction of rat brain cDNA, the present study involved the sacrifice of one rat Wistar. The rat was sacrificed by decapitation according to the ethical guidelines and the procedures were reviewed and approved by the Ethics Committee of Federal University of São Paulo (from Portuguese, Universidade Federal de São Paulo–UNIFESP, Proc. 1001/09).

### Cygb Purification

Soluble His6-tagged Cygb was purified from the lysates using a four-stage process of fast protein liquid chromatography (AKTA purifier, GE) composed of affinity column (Histrap FF, GE), a desalting step (Hitrap desalting, GE), size exclusion chromatography (Superdex 75, GE) and another desalting column prior to lyofilization. In all steps, Cygb was spectrometrically followed through 415 nm corresponding to the Soret band. Briefly, after 25 μm filtration and 1:5 dilution into 20 mmol.L^-1^ phosphate 250 NaCl pH 7.4, the protein solution was eluted from the affinity column against the same buffer containing an imidazol gradient. Both desalting and size exclusion chromatrography were conducted using a 20 mmol.L^-1^ phosphate 250 mmol.L^-1^ NaCl pH 7.4 buffer. The last desalting step was performed in a 5 mM phosphate pH 7.4 buffer. The resulting purified Cygb was tested for purity in a Commassie Blue-stained 12% SDS-PAGE. The product of last step was lyophilized in aliquots and stocked at -20°C. The aliquots were resuspended in milliQ water prior to use. All experiments were conducted using fresh expressed and purified Cygb to avoid undesirable oxidative reactions and all lots were carefully analyzed to guarantee homogeneity. Inter-batch replicates were conducted in all spectroscopic experiments, but only representative results are shown here.

### Electronic absorption measurements and analysis

Ultraviolet (UV)-visible spectra were obtained in a NanoDrop Spectrophotometer ND-100 (Nanodrop Technologies, Wilmington, DE, USA). The reactions were realized in buffer phosphate 20 mmol.L^-1^, pH 7.4, at 37°C, in incubations of 0, 10 and 60 min. The experiments of electronic absorption spectroscopy were performed with 65 μmol.L^-1^ Cygb using 0.1 cm optical length. When present, the concentration of peroxides was 650 μmol.L^-1^.

### Circular Dichroism and Magnetic Circular Dichroism measurements and analysis

Circular dichroism measurements were carried out, at room temperature, using a Jasco J-720 spectropolarimeter (JASCO Inc.Easton, MD, USA), a 0.1 cm quartz cuvette. The parameters were: a bandwidth of 1.0 nm, a scanning speed of 200 nm/min, a response time of 0.25 s and 4 accumulations. Two wavelength ranges were used depending on the chromophore to be analyzed: the protein structure or the prosthetic group, heme iron. For the secondary structure determination, a 20 μmol.L^-1^ protein solution in 20 mmol.L^-1^ phosphate buffer, pH 7.4 was measured in the far-UV wavelength range (250–190 nm) and recorded as an average of 4 scans at 200 nm min^-1^ using a response time of 0.25 s. Secondary structural predictions were done using the CDPro software package [44–46]. For the analysis of heme iron, a 20 μmol.L^-1^ protein solution in 20 mM phosphate buffer, pH 7.4, was measured in the near UV-visible range (700–300 nm). Spectra were recorded as an average of 4 scans at 200 nm min^-1^, collecting data points every 1 nm, in the presence of variable magnetic fields provided by an attached electromagnet set in the positive and negative mode. The maximal magnetic field used was 0.995 T. The MCD spectral component on each magnetic field was determined by subtracting the spectra run in the positive and negative modes of the electromagnet. The corresponding CD spectral component was obtained from the sum of the spectra run in the positive and negative modes of the electromagnet.

### EPR measurements

Direct continuous wave EPR of Cygb heme iron was measured as previously described for other hemeproteins with modifications [47,48]. The protein concentration was of 1.2 mmol.L^-1^ and when present, the peroxide concentration was of 12 mmol.L^-1^. The EPR measurements were carried out using an EPR Bruker system, the ELEXSYS E580 model, equipped with a helium cryostat (Oxford, UK) and a temperature controller under the following conditions: a central field of 240 mT, a scanning field of 400 mT, 2048 points, a modulation amplitude of 1 mT, a gain of 45 dB, a temperature of 11 K, a time constant of 20.48 ms, a conversion time of 81.92 ms and a microwave power of 5 mW. After the addition of the reactants in different media, 120 μL of the mixture was quickly introduced into an EPR quartz tube, cooled in liquid nitrogen and transferred to the helium cryostat assembled in the EPR cavity to obtain the spectra.

Detection of tyrosil radicals in peroxide-challenged Cygb was conducted using DBNBS spin trapping, as previously described in the literature [47]. The EPR spectra were measured using 20 μmol.L^-1^ protein solution, 1 mmol.L^-1^ hydrogen peroxide and 20 mmol.L^-1^ DBNBS. The radical adduct was measured using an X-band EPR Varian system, the E-109 model. The EPR conditions were: a microwave frequency of 9.5077 GHz, a central field of 340 mT, a scanning field of 16 mT, 1024 points, a modulation amplitude of 0.05 mT, a gain of 5.0 × 10^5^, a temperature of 293 K, a time constant of 0.128 s, a scan time of 180 s and a microwave power of 20 mW.

EPR spectra of the Cygb^•^-DBNBS adduct were simulated with NLSL software using the following simulation parameters: gyromagnetic 4-* and hyperfine 4-* tensors, an isotropic Gaussian line width (gib0) and parameters related to system ordering (S0 and S2) and dynamics (4* tensor). The large number of parameters that must be minimized makes the simulation process more difficult and complex. The simulation was carried out using typical values of gyromagnetic and hyperfine tensors for nitroxides with the hyperfine isotropic split value fixed at 1.36 mT. From three tentative simulations, the best fit was obtained by aligning the yR axis with the local director zd, assuming the Euler angles of the system of rotational diffusion to be βR = 90° and γR = 90°. The correlation time related to the rotational diffusion tensor was on the order of 10^−7^ s, consistent with the protein rotation rate. The measurements were repeated after Cygb digestion by 10 mg.mL^-1^ proteinase K [49,50].

### Epifluorescence microscopy

Microscopy images of the fibrils were obtained using a wide field Leica DMI 6000B microscope (Leica Microsystems, Germany) with a HCX PL APO 100×/1.30 objective coupled to an ultrafast digital camera (Leica DFC365 FX, Leica Microsystems, Germany) with a I3 filter cube (excitation filter BP 450–490, suppression filter LP 515). For the epifluorescence experiments 70 μmol.L^-1^ protein solution was incubated for 1 h with 700 μmol.L^-1^ peroxide solution in the presence of thioflavin-T used for fibril dying.

### Low-vacuum Scanning Electronic Microscopy

The scanning electron microscopy (SEM) of the samples was carried out using a compact low vacuum electronic scanning microscopy JSM-6010LA, JEOL (Tokyo, Japan). For the low-vacuum SEM experiments, it was used 7 μmol.L^-1^ cygb solution with 70 μmol.L^-1^ peroxide solutions. When present, glutathione was equimolar or tenfold molar excess relative to the protein concentration.

### Fourier Transformer Infrared (FTIR) Spectroscopy

FTIR spectra were recorded with a Perkin Elmer Spectrum RX1 FTIR spectrometer with a resolution of 4 cm^−1^. For FTIR measurements, 7 μmol.L^-1^ protein solution was incubated with 70 μmol.L^-1^ peroxide solutions for 1 h. After the incubation some fibrillary aggregates precipitated at the bottom of the vial and they were visible even to the naked eyes. These precipitated fibrils were collected with an automatic pipette and analyzed by FTIR.

### Physical protein-protein interaction and protein-chemical interaction network design and global topological analysis

Hydrogen peroxide and Cygb classically described in the literature as being related to oxidative stress were the key words used to obtain information about interactions in the context of physical protein-protein interaction (PPPI) networks and protein-chemical interaction (PPCI) networks. In this sense, the data mining screening and network design of PPPI and PPCI networks were performed in STRING [http://string.embl.de/] and STITCH [http://stitch.embl.de/] using the following parameters: all active prediction methods enabled except text mining, no more than 50 interactions, a high confidence score (0.700) a network depth equal to 2 and other default parameters. The data were analyzed using Cytoscape software, version 2.8.3 [51]. PPPI networks obtained from this first screening were then combined in a unique PPCI network by employing the union function of the Cytoscape core plugin Merge Networks. The union PPCI network was then analyzed using molecular complex detection (MCODE) software [52], a Cytoscape plugin (http://apps.cytoscape.org/apps/mcode) in order to detect clusters of proteins that could represent distinct biologic processes. The parameters used for MCODE to generate the sub networks were as follows: loops included, a degree cutoff of 2, deletion of single connected nodes from the cluster (haircut option enabled), expansion of the cluster by one neighbor shell allowed (fluff option enable), a node density cutoff of 0.1, a node score cutoff of 0.2, a k core of 2, and a maximum depth of network of 100. The generated protein and chemical networks were further studied by focusing on the major biological associated processes using Biological Network Gene Ontology (BiNGO) [53] software. The degree of functional enrichment for a given cluster and category was quantitatively computed (P value) using hypergeometric distribution [54], and a multiple test correction was also assessed by applying the false discovery rate algorithm [55], which was fully implemented through the BiNGO software with a significance level of 0.05.

## Results and Discussion

### Spectroscopic characterization of the recombinant Cygb revealed the predominance of the resting-form, ferric, low-spin hemeprotein

**Fig 1:**
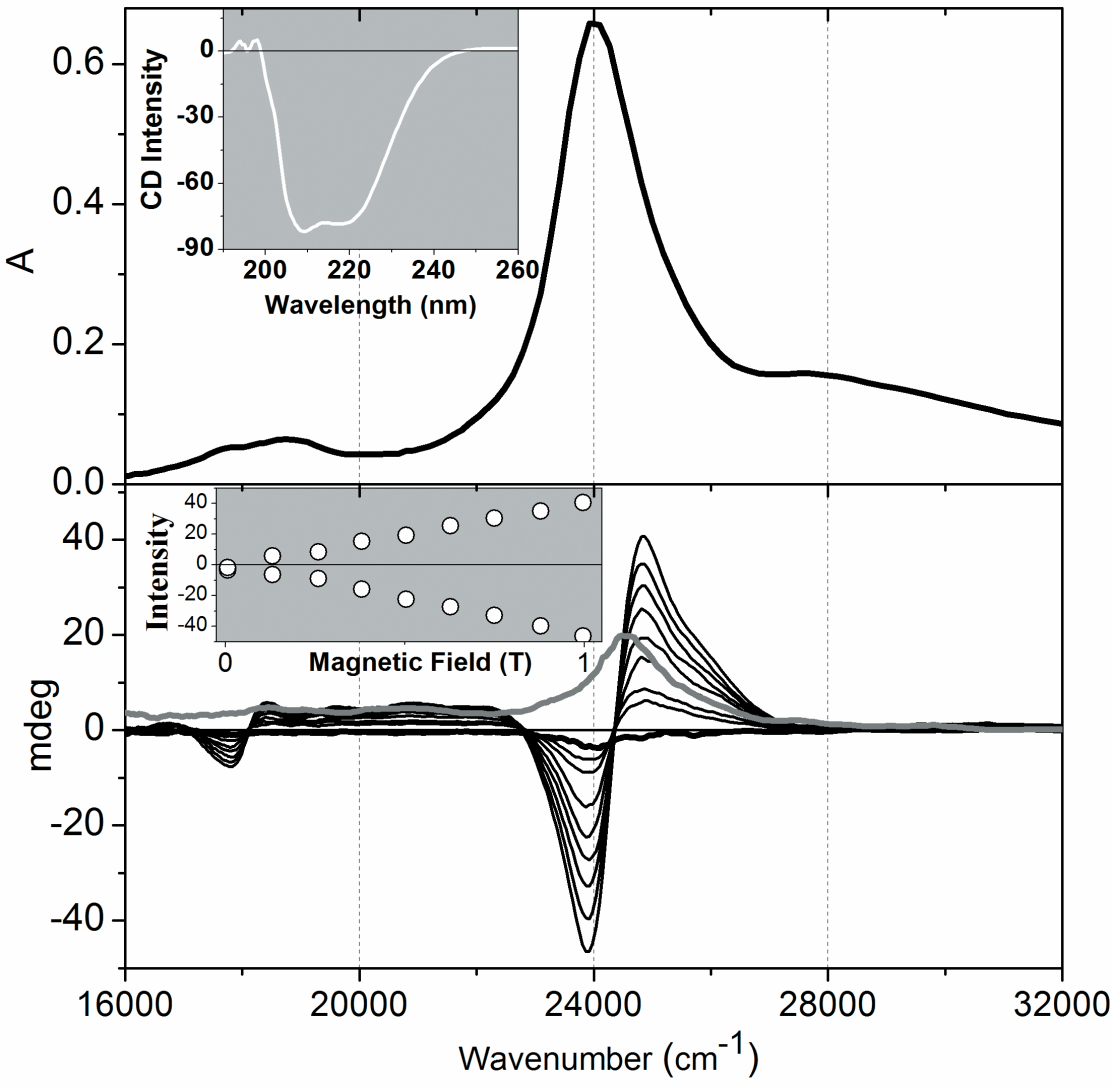
Spectroscopic characteristics of Cygb. The upper panel shows the EA spectrum of Cygb and the respective inset the corresponding far-UV CD spectrum. The lower panel shows the CD (light gray line) and the MCD spectra of Cygb obtained by addition and subtraction of the original spectra obtained at positive and negative magnetic fields. MCD is shown at increasing magnetic fields and the respective inset shows the linear increase of Soret band intensity promoted by increasing the magnetic field. The experiments of EA spectroscopy were performed with 65 μmol.L^-1^ Cygb using 0.1 cm optical length. The experiments of CD and MCD were performed using 20 μmol.L^-1^ protein solution in 20 mmol.L^-1^ phosphate buffer, pH 7.4. These results are representative of three independent replicates.

The resting form of a recombinant Cygb was characterized by UV-visible electronic absorption (EA), far-UV CD, visible CD, MCD and EPR at the temperature of helium liquid. The results are shown in Fig 1: UV-visible EA and far-UV CD (upper inset), visible CD (light gray line) and MCD spectra of Fe^3+^ Cygb at increasing magnetic field values (black lines). The far-UV CD spectra for native Cygb had negative bands at 208 nm and 222 nm (inset of Fig 1), consistent with a high percentage of α-helice. Secondary structure prediction was generated using CDPro.12-13 and the α-helice content was estimated to be 72%. This high value is comparable to the α-helice content of the members of the globin family such as Mb (76%) or the α-helice content of highly helical proteins such as the four-helice bundle (>80%) [56,57]. The EA, visible CD and MCD data are plotted using a wavenumber scale, enabling a comparison of the energy separations between the major spectral bands of the hemeprotein. The optical spectrum of Fe^3+^ Cygb is essentially identical to that previously described in the literature. As expected for a hemeprotein, Fe^3+^ Cygb exhibits a Soret band that results from the overlap of the π-π* and π-*d* transitions that can be also seen in the MCD spectrum. At lower energies, it is observed the almost fully forbidden Q band. The much lower absorption intensity of the Q band results from the accidental near degeneracy of the HOMOs (highest occupied molecular orbital). The Q band gains intensity from the allowed Soret (*B*) band through a vibrational borrowing mechanism [58]. The Soret MCD band of Fe^3+^ Cygb features a derivative pattern, but the zero crossing slightly deviates from the UV-visible Soret band peak (24000 cm^-1^). This feature results from the contribution of the Faraday *B*-term to the Soret MCD band (π-π* transition). The presence of the Faraday *B*-term is indicative of the mixing of an intermediated (ǀK>) state with the excited (ǀJ>) state promoted by the magnetic field. The presence of a Faraday *A*-term associated with the CT (charge transfer) band indicates the presence of degenerate *d* orbitals that are split by the magnetic field [59,60].The lower inset of Fig 1 shows the linear increase in positive and negative MCD Soret bands with increasing magnetic field. The CD spectrum was obtained by the addition of positive and negative MCD spectra at the maximum magnetic field applied (0.995 T); MCD spectra with duplicated intensity were obtained by subtracting the negative bands from the positive bonds.

**Fig 2:**
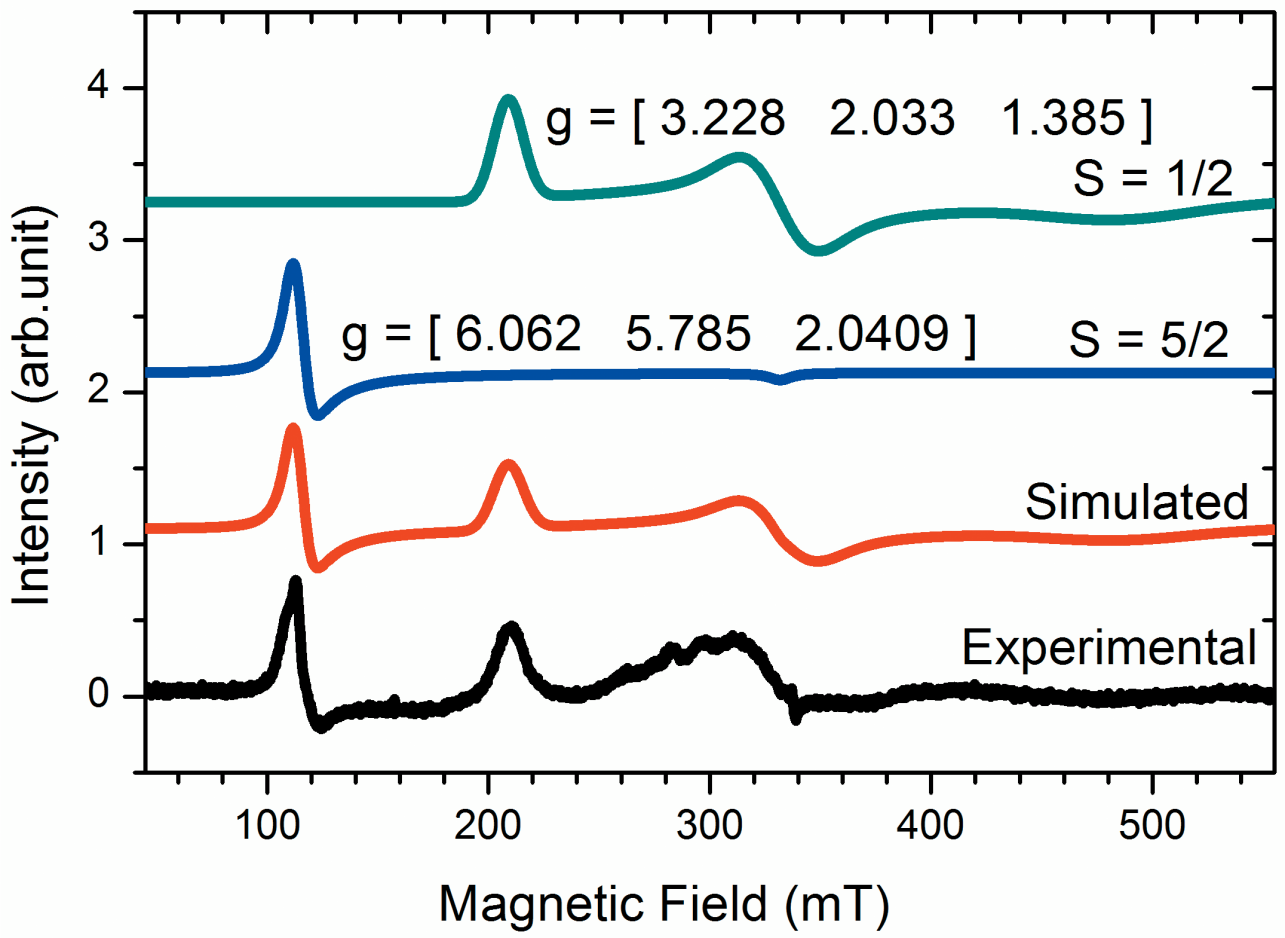
EPR spectrum of resting Cygb and the spectral components obtained by simulation. The red line corresponds to simulation of the composite spectrum and the blue and green lines correspond to the high and low spin components, respectively. The g values of the low spin state component are: g1 = 3.228, g2 = 2.033 and g3 = 1.385 with rhombic distortion and for the high spin state the g values are: g1 = 6.062, g2 = 5.785 and g3 = 2.0409. The simulation was done by the software Symphonia. For EPR experiments, the protein concentration was of 1.2 mmol.L^-1^. These results are representative of three independent replicates.

All the spectra shown in Fig 1 are typical of native Fe^3+^ Cygb with bis-histidine hexacoordinated heme iron that was corroborated by CW-EPR analysis (Fig 2). Fig 2 shows the EPR spectrum of resting Cygb and the spectral components obtained by simulation. The EPR spectrum of resting Cygb reveals that the heme iron is predominantly in the native low spin state with g_1_ = 3.228, g_2_ = 2.033 and g_3_ = 1.385 and rhombic distortion. The sample furthermore contains a low concentration of the protein in the high spin state with g values g_1_ = 6.062, g_2_ = 5.785 and g_3_ = 2.0409. The EPR signal of heme iron in the high spin state is very intense relative to the low spin state and very low concentrations of the hemeprotein in the high spin state are sufficient for intense signal detection [60].

### Cygb exhibits peroxidase activity with a mechanism similar to that described for Mb

**Fig 3:**
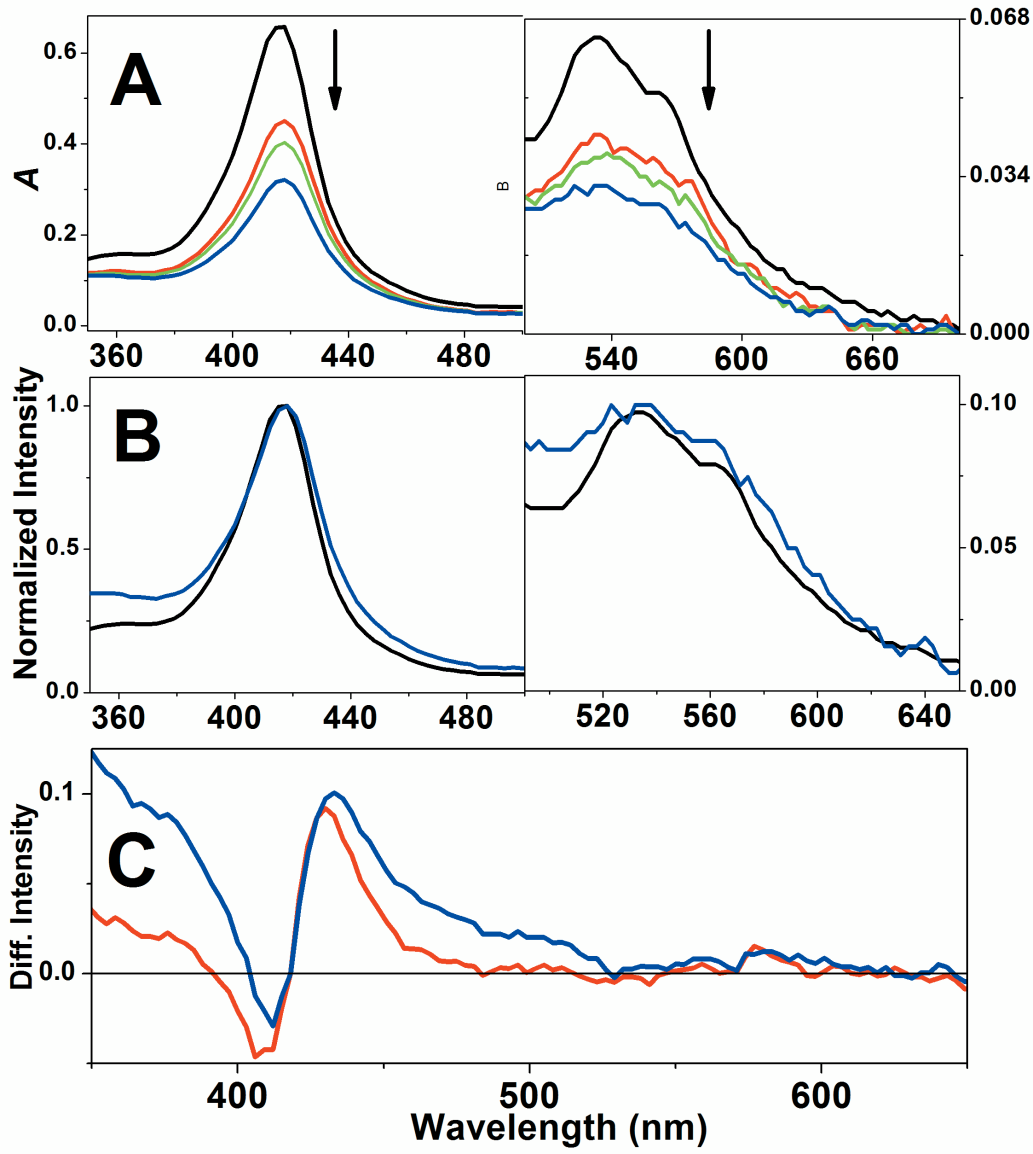
Changes in the EA spectrum of Cygb during the reaction with hydrogen peroxide. A) Bleaching of Soret and Q bands of EA spectra of Cygb in the course of the reaction with hydrogen peroxide. The black line represents the EA spectrum of resting Cygb, red, green and blue lines corresponds to the spectra obtained at 30, 60 and 200 s after addition of hydrogen peroxide and indicated by the arrows. B) Normalized spectra of Cygb resting form and 200 s after hydrogen peroxide addition. C) Differential spectra of Cygb obtained 30 and 200 s after the addition of hydrogen peroxide The experiments of EA spectroscopy were performed using 65 μmol.L^-1^ Cygb and 0.1 cm optical length. When present, the concentration of peroxide was 650 μmol.L^-1^. These results are representative of three independent replicates.

Native Cygb was challenged by three types of peroxides: hydrogen peroxide, *t-*BuOOH and CumOOH and the reactions were analyzed using EA and EPR spectroscopy. Fig 3A shows that hydrogen peroxide promotes progressive bleaching of the Cygb Soret and Q bands. However, the normalization of the Cygb spectra (Fig 3B) that were run during the reaction with hydrogen peroxide revealed a red shift of the Soret band (from 416 to 418 nm) 30 sec after the start of the reaction. The bleaching also occurred with the Q band without a significant red shift. The occurrence of a Soret band red shift is consistent with the expected formation of Compound II. The slight red shift can be realized via an analysis of the Cygb heme iron coordination sphere. Cygb has a hexacoordinated heme iron with a strong field ligand, histidine, at the sixth coordination position. This heme iron coordination sphere responds to the red shifted Soret band relative to the pentacoordinated hemeproteins and the 2 nm redshift after the Cygb conversion to Compound II in which a double-bounded oxygen is the sixth ligand of heme iron (Fe^4+^ = O).

**Fig 4:**
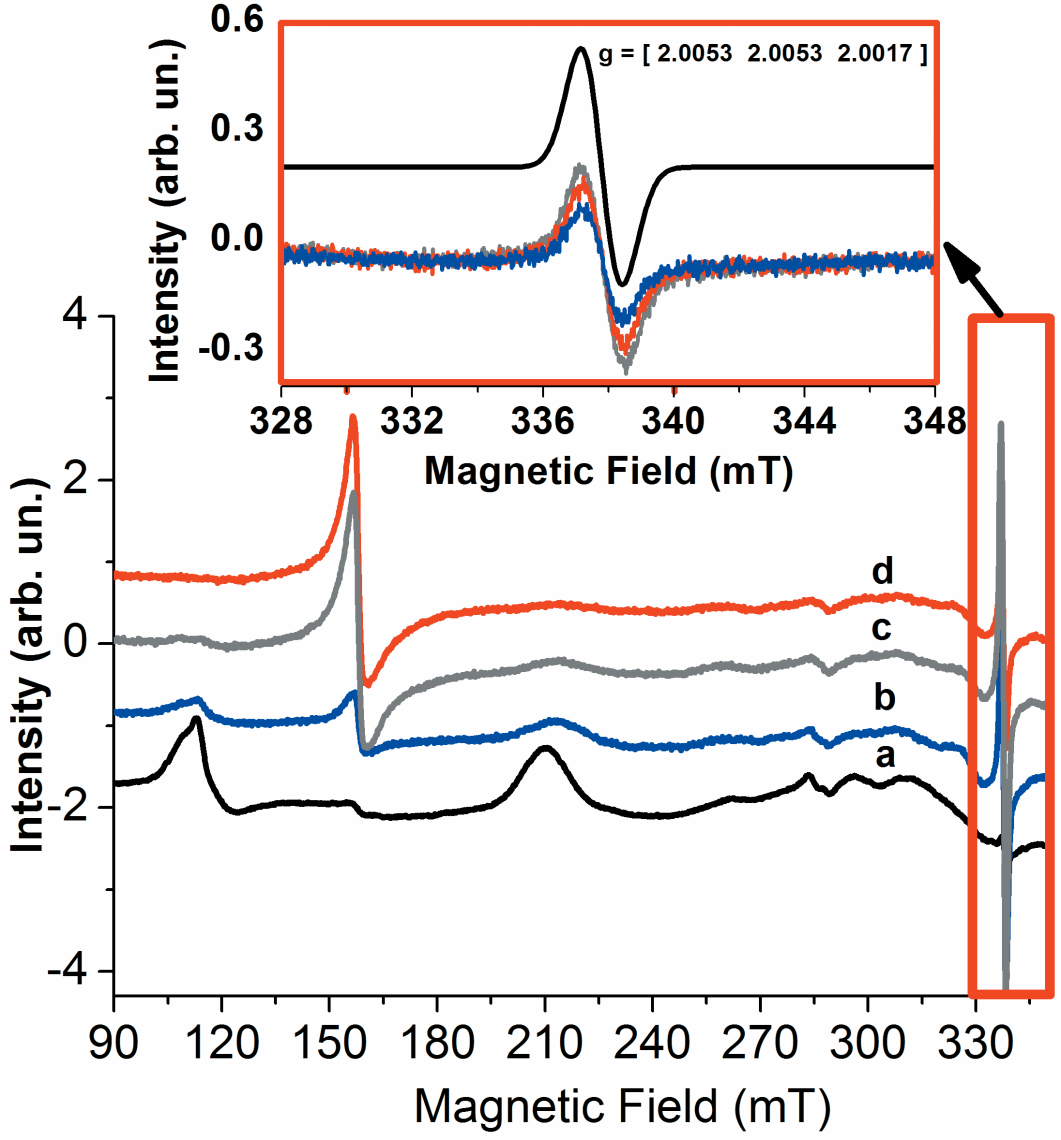
Changes in the EPR spectrum of resting Cygb during the reaction with hydrogen peroxide. The spectra marked as a, b and c were obtained at 30, 60 and 210 s after the addition of hydrogen peroxide. The inset shows a zoom in the spectra of the free radical produced concomitantly with the formation of high valence species. For EPR experiments, the protein concentration was of 1.2 mmol.L^-1^and when present, the peroxide concentration was of 12 mmol.L^-1^. These results are representative of three independent replicates.

In the following, the formation high valence states of Cygb during the reaction with hydrogen peroxide was investigated by CW EPR of Cygb heme iron (Fig 4). Fig 4 shows the changes in the Cygb EPR spectrum run during the reaction of hydrogen peroxide with Cygb. The addition of hydrogen peroxide promotes a decrease in the EPR signal of the resting form of Cygb. This result is consistent with the formation of the EPR silent oxoferryl form of heme iron (Fe^4+^ = O). In addition, we observed a progressive increase in the signal with g = 4.3 that might be due to oxidative damage of heme iron [59], also supported by the occurrence of Soret band bleaching in the EA spectrum (Fig 3A). In the course of the reaction of Cygb with hydrogen peroxide, we also observed the signal of a free radical with g_1_ = 2.0053, g_2_ = 2.0053 and g_3_ = 2.0017. The EPR parameters are consistent with a tyrosyl radical, as has also been described for other hemeproteins such as cytochrome c oxidase [61,62]. The g*y* and g*z* values that were determined for the protein-centered radical are according to the known tyrosyl radicals. These values are in general equal to or larger than 2.0042 and 2.0020, respectively. The g*x*-g*y* and g*x*-g*z* values are equal to 0.0036, which is close to the range described for tyrosyl radicals (0.007–0.004) and close to the value described for Micobacterium tuberculosis catalase-peroxidase (0.00364) [63].

**Fig 5:**
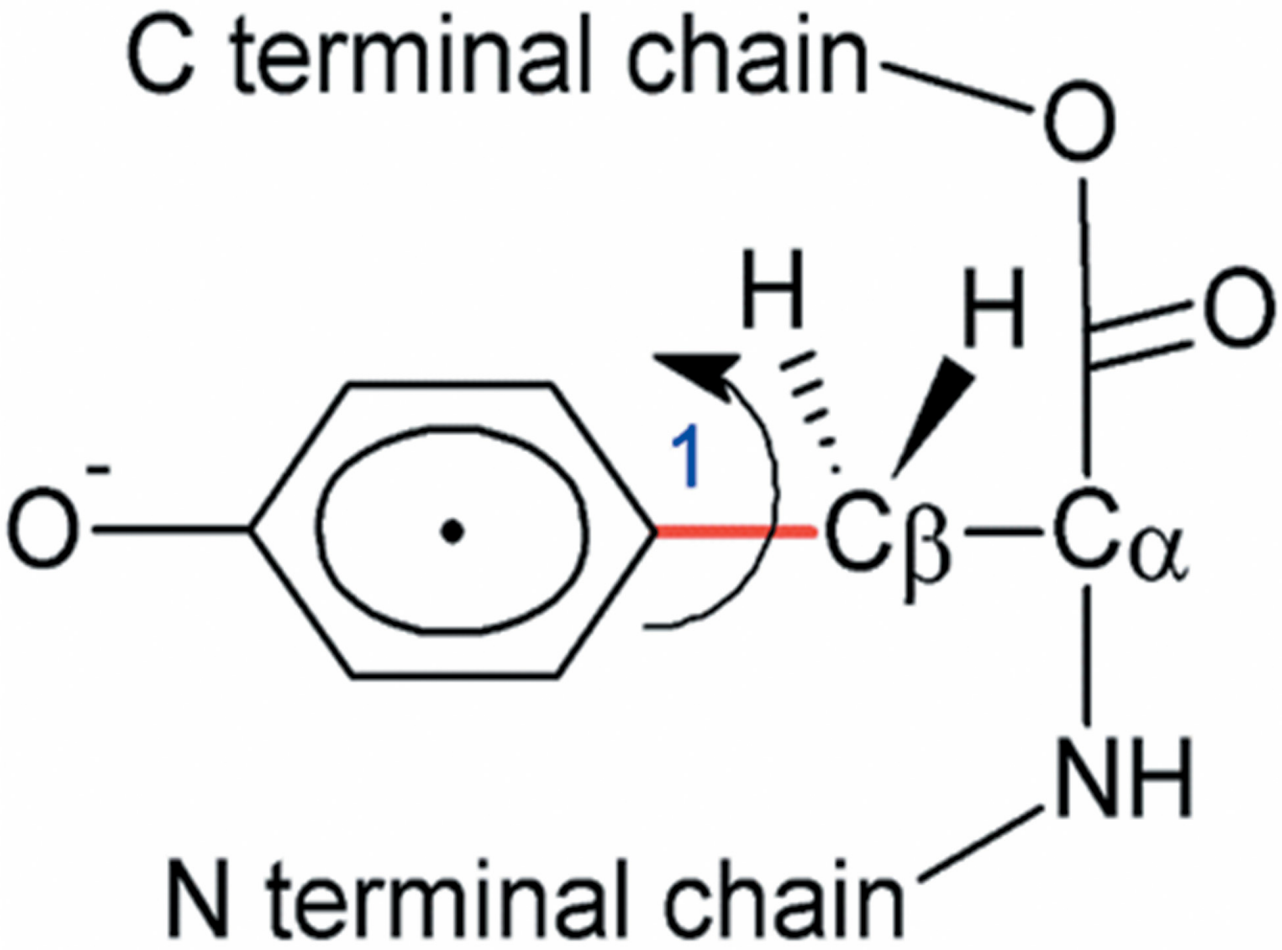
Structure of tyrosyl radical in a protein polypeptide chain. In the structure it is indicated the rotation of phenoxyl radical around carbon β.

The tyrosyl radical has an unpaired electron delocalized over the π ring and its EPR signal is determined by the hyperfine interaction with the four ring protons (Fig 5). Considering that the phenol group in the tyrosine structure can rotate around the C_β_-C1 bond, the EPR spectra of tyrosyl radical in proteins are extremely variable because of the different microenvironments caused by the protein chains [61,63,64].

**Fig 6:**
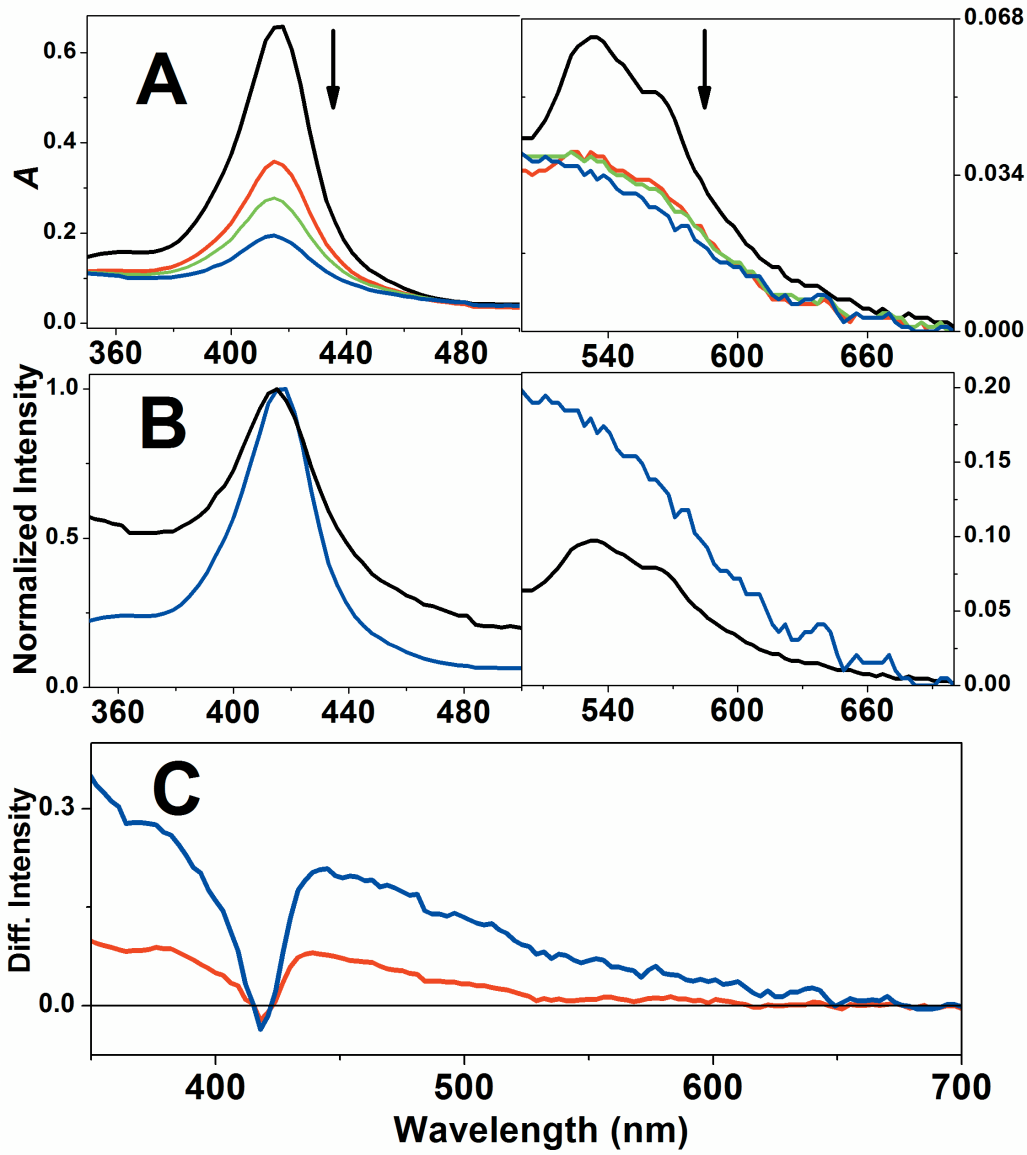
Changes in the EA spectrum of Cygb during the reaction with *t*-BuOOH. A) Bleaching of Soret and Q bands of EA spectra of Cygb in the course of the reaction with *t*-BuOOH. The black line represents the EA spectrum of resting Cygb, red, green and blue lines corresponds to the spectra obtained at 30, 60 and 200 s after addition of *t*-BuOOH and indicated by the arrows. B) Normalized spectra of Cygb resting form and 200 s after *t*-BuOOH addition. C) Differential spectra of Cygb obtained 30 and 200 s after the addition of *t*-BuOOH. The experiments of EA spectroscopy were performed using 65 μmol.L^-1^ Cygb and 0.1 cm optical length. When present, the concentration of peroxides was 650 μmol.L^-1^. These results are representative of three independent replicates.

Fig 6 shows the spectral changes in the Cygb EA spectrum of resting Cygb challenged by *t*-BuOOH. The spectral changes in the Cygb EA spectrum during the reaction with *t*-BuOOH differed from those observed when hydrogen peroxide was used as a reagent. *t-*BuOOH promoted more intense bleaching of the Soret band and a 1.5 nm blue shift was observed for the Soret band. The Q band also exhibited significant bleaching and the normalized spectra revealed a significant temporal increase of the 460/550 nm absorbance ratio that was not observed when hydrogen peroxide was used as the substrate.

**Fig 7:**
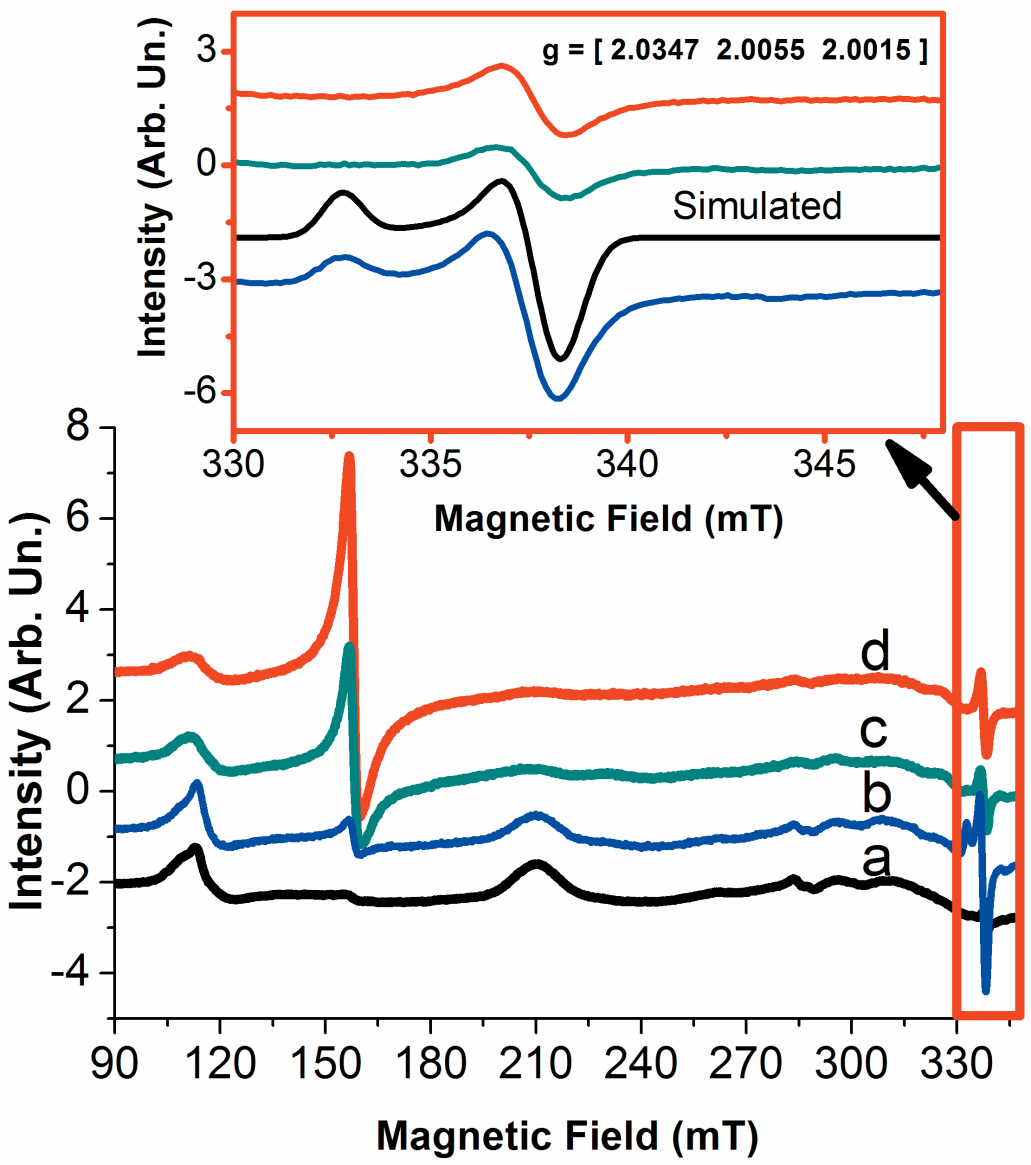
Changes in the EPR spectrum of resting Cygb during the reaction with *t*-BuOOH. The spectra marked as *a*, *b* and *c* were obtained at 30, 60 and 210 s after the addition of *t*-BuOOH. The inset shows a zoom in the spectra of the free radical produced concomitantly with the formation of high valence species. For EPR experiments, the protein concentration was of 1.2 mmol.L^-1^and when present, the peroxide concentration was of 12 mmol.L^-1^. These results are representative of three independent replicates.

CW-EPR of Cygb heme iron during the reaction with *t-*BuOOH (Fig 7) showed a progressive decrease in the low spin signal, with g values of 3.228, 2.033 and 1.385. Interestingly, the *t*-butylperoxyl free radical that had been previously observed during the reaction of cytochrome c with *t*-BuOOH was detected at the early times of the reaction (30 sec) in which almost 80% of the low-spin signal was yet present [65,66]. The *t*-butylperoxyl radical and the heme iron low spin signals disappeared at 60 sec, concomitantly with a significant increase of the g = 4.3 signal and the appearance of the same signal of a protein-centered radical that was detected during the reaction with hydrogen peroxide. The intensity of the protein radical signal increased at 210 sec. Very similar EA and EPR results were obtained for the reaction of Cygb with CumOOH. In this condition, the Soret band blueshift was observed in the EA spectrum, as well as a protein-centered free radical in the EPR spectrum (not shown).

**Fig 8:**
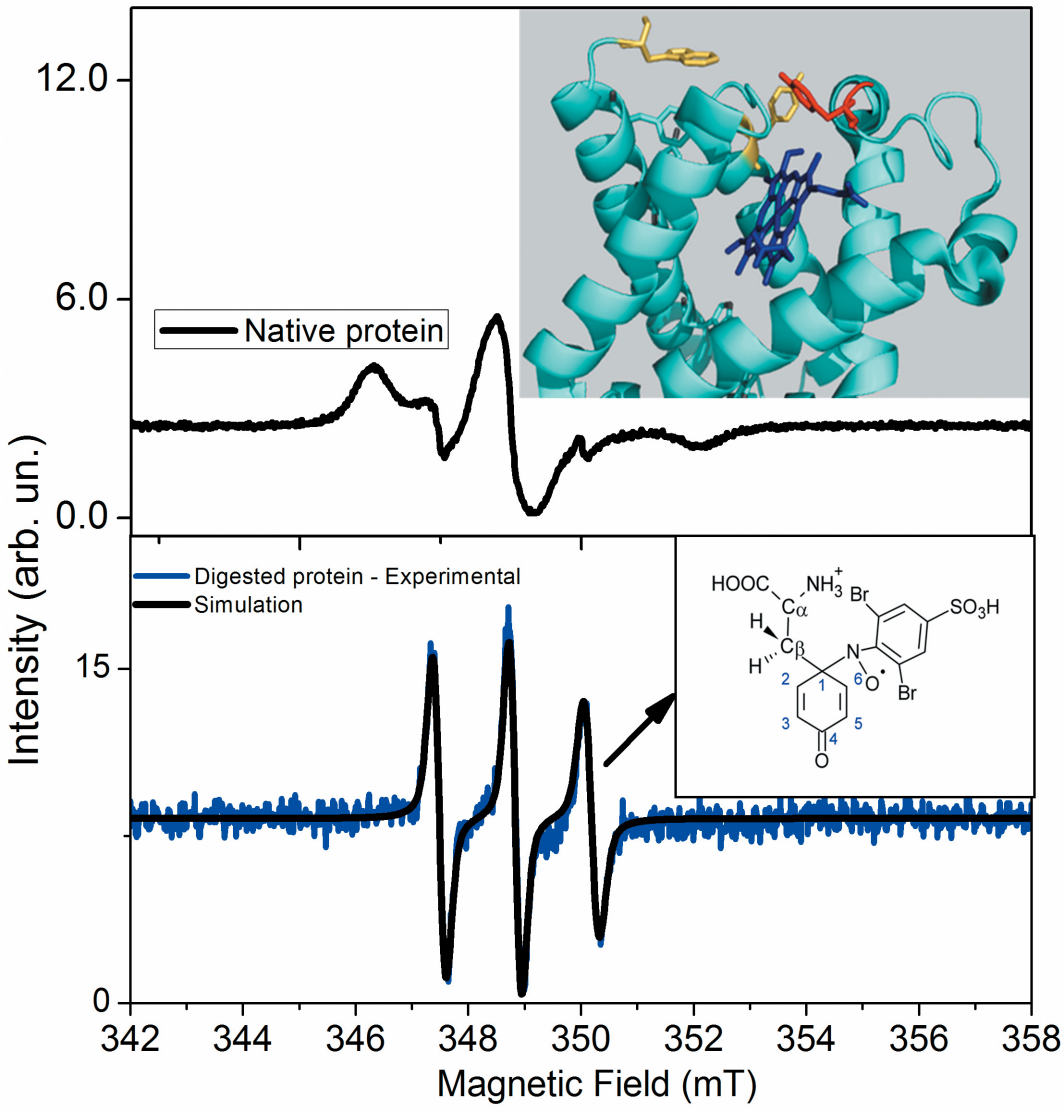
EPR spectra of tyrosyl DBNBS-adduct immobilized in Cygb challenged by hydrogen peroxide. The upper panel shows the EPR spectrum of tyrosyl DBNBS adduct in the native protein and the lower panel shows EPR spectrum of tyrosyl DBNBS adduct after digestion by proteinase K. The simulation of the EPR spectra of DBNBS adduct was performed as described in materials and methods. The simulation parameters obtained for this spectrum were: A_N_ = 13.5 Gauss, g = 2.0061, L/G = 0.2 and LW = 2.40 Gauss, considering the tumbling effect (LW = *a* + *b* x *m* + *c* x *m* ^2^ and *a* = 2.3 *b* = -0.2 and *c* = 0.3128). The EPR parameters are consistent with tyrosyl-DBNBS adduct. The inset of upper panel shows a zoom of heme region in the tertiary Cygb structure. The residue represented in red corresponds to Tyr59. Tyr131 and Trp123 are represented in yellow. The inset of the lower panel shows the structure of a C-1-centered radical of tyrosine phenoxyl ring. The EPR spectra were measured using 20 μmol.L^-1^ protein solution, 1 mmol.L^-1^ hydrogen peroxide and 20 mmol.L^-1^ DBNBS. These results are representative of three independent replicates.

To corroborate the identity of the protein-centered free radical detected by direct CW-EPR measurements at low temperature, we also analyzed the free radical using an EPR spin trapping technique. The formation of the tyrosyl radical during the reaction of Cygb with hydrogen peroxide is related to the mechanism of the peroxide cleavage. It is expected that the reaction of Cygb with hydrogen peroxide converts the heme group to oxo-ferryl π cation species. However, considering the similarity of the Cygb structure with that of Mb, it is possible that the oxidizing equivalent of the porphyrin ring is transferred to a globin amino acid residue. In this case, it is more likely that the oxidation of tyrosine 59, which is positioned close to the vinyl, methyl edge of the heme group, occurs. The tyrosine residue 123 must not be discarded because it is also in the proximity of the edge of the heme group. Tryptophan 171 is farther from the heme group and could be a secondary location of the radical, more likely by the transfer of one oxidant equivalent of a tyrosyl radical. DBNBS added to a Cygb solution challenged by hydrogen peroxide provided an EPR spectrum consistent with that of a partially immobilized nitroxide (Fig 8, upper panel). EPR signals were not detected when any of the reactants was absent (not shown). The EPR spectra of the DBNBS adduct was simulated, as described in the Materials and Methods section. The simulation resulted in a large Gaussian line width (3.77 G). For this tyrosyl-DBNBS adduct, the large linewidth results from the super hyperfine interactions in a highly heterogeneous microenvironment around nitroxide radical; these interactions were not resolved by the CW EPR spectrum [47]. The oxidative potential of tyrosine and tryptophan are similar; when these residues are neighbors in a protein structure, the unpaired electron density can be found in these amino acids in a population of hemeproteins treated with peroxides. However, considering the EPR parameters of the direct EPR signal and the location of tyrosine 59 and 123 at the vicinity of heme group edge (inset of Fig 8, upper panel), it is reasonable to assign the location of the unpaired electron to a tyrosine residue [49,67]. When DBNBS was added to the Cygb solution challenged by hydrogen peroxide and the sample underwent nonspecific proteolysis by proteinase K, an isotropic ESR spectrum consisting primarily of three lines was detected (Fig 8, lower panel). The simulation parameters obtained for this spectrum were: A_N_ = 13.5 G, g = 2.0061, L/G = 0.2 and LW = 2.40 G, considering the tumbling effect (LW = *a* + *b* x *m* + *c* x *m* ^2^ and *a* = 2.3, *b* = -0.2 and *c* = 0.3128). The EPR parameters are consistent with a tyrosyl-DBNBS adduct. The EPR spectrum does not exhibit additional hyperfine structure that is consistent with a tertiary carbon-centered radical adduct establishing no bonds with atoms with nuclear spin. Such a structural feature is consistent with the C-1-centered radical of a tyrosine phenoxyl ring (inset Fig 8, lower panel).

Considering, the papers of Detweiler et al [68] and Lardinois et al [69], we tried to identify by MS/MS analysis, the tyrosine residue that is converted to a free radical intermediate during the reaction of Cygb with peroxides. These authors report the identification of tyrosines of myoglobin and neuroglobin, respectively using MS/MS analysis of a DMPO-protein adduct. For unknown reasons, DMPO failed to trap Cygb tyrosil radical. Thus, EPR spin trapping signal of DMPO adducts and changes in the Cygb mass were not detected when Cygb was incubated with hydrogen peroxide in the presence of DMPO. DBNBS efficiently trapped Cygb tyrosil radical (Fig 8) and the MS analysis of Cygb incubated with hydrogen peroxide and DBNBS revealed a population of protein with mass increase of 777.6 Da that is compatible with the presence of two Na^+^DBNBS adducts (not shown). However, the MS spectrometry of the native Cygb and of Cygb-DBNBS digested by trypsin did not yield the fragments that contain tyrosine 59 and 123, the most probable sites of the free radical (not shown). Although the inconclusive result obtained for the tryptic fragments of Cygb, the mass increase of 777.6 Da compatible with the presence of two Na^+^DBNBS adducts is consistent with tyrosine residues 59 and 123 as the most probable sites of tyrosil radicals. The possibility of radical transfer from cytochrome c to recipient peptides was demonstrated by Deterding et al [70]. Therefore, for Cygb, it is possible that the tyrosil radical formerly generated in tyrosine 59 after a peroxide cleavage, could be rapidly transferred to the neighbor tyrosine 123. The free radical could than to be regenerated in tyrosine 59 again after the cleavage of a new peroxide molecule. Therefore, two tyrosil radicals could be generated in a Cygb molecule and to be trapped by DBNBS. Experiments to identify these tyrosines by site-directed mutagenesis are started in our laboratory.

### Oxidative damage of Cygb leads to the formation of amyloid fibrils

**Fig 9:**
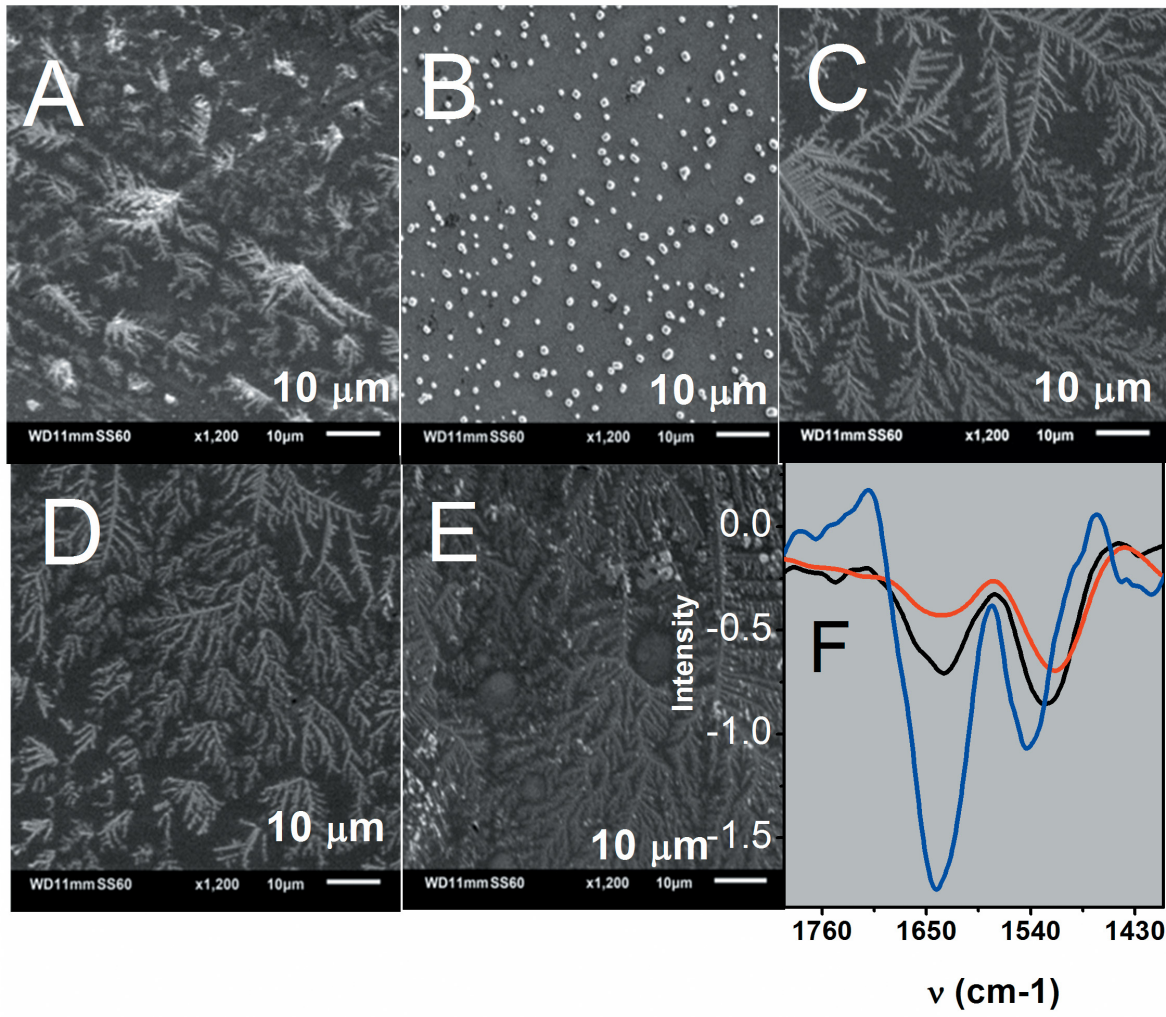
Formation of Cygb amyloid structure after challenge by peroxides. A) Low-vacuum SEM image of native Cygb. B) Low-vacuum SEM image of native Cygb in the presence of 10 fold molar of GSH. C), D) and E) Low-vacuum SEM image of Cygb challenged by hydrogen peroxide, t-BuOOH and CuOOH, respectively. For SEM images the samples were dryed on a silicon surface by the purging N_2_. Panel F shows the FTIR spectrum of Cygb films obtained by partial drying of Cygb solutions by N_2_ purging. The black, blue and red lines correspond to native Cygb, presence of GSH and presence of *t*-BuOOH.

The incubation of Cygb with peroxides leads to the appearance of some precipitates on the bottom of the tubes that could be caused by the formation of amyloid structures. The native protein in aqueous buffered solution was dried at room temperature by purging N_2_ and then analyzed by low vacuum scanning electronic microscopy. The same procedure was performed for the samples previously incubated with peroxides. Fig 9A shows that the native protein formed protein fibrils when dried by N_2_ purging and in the presence of air. In these conditions, the Cygb fibrils resemble pine leaves with short branches. To determine whether the formation of fibrils was favored by the oxidation of the protein during drying, the protein was treated with an equimolar amount of GSH (not shown) and also with a 10-fold excess of the antioxidant (Fig 9B). Microscopy images reveal that GSH prevented the formation of fibrils. In the presence of an equimolar amount of GSH, the 300× magnification image showed that the protein aggregates yielded a pattern of truncated brunches (not shown). The pattern of truncated brunches disappeared when the samples were treated with a 10-fold excess of GSH and the dried protein formed globular aggregates (Fig 9B). The samples of hydrogen peroxide-treated Cygb showed a significant increase in the amount and length of the protein fibrils looking like pine branches (Fig 9C). The treatment of the protein with t-BuOOH and CumOOH led also to the formation of fibrils similar to those observed for the samples treated with hydrogen peroxide (Fig 9D and 9E, respectively). The formation of amyloid fibrils of oxidized Cygb was also corroborated by FTIR (Fourier transform infrared) analysis (Fig 9F). In the FTIR spectrum of air-oxidized and t-BuOOH-oxidized Cygb, we observed a shift of the amide I band from 1640 to 1631 cm^-1^ that is consistent with the change of native α-helical structure to stacked β-sheets [71,72]. The samples of Cygb incubated with organic peroxides, particularly with CumOOH, yielded a higher density of fibrils.

**Fig 10:**
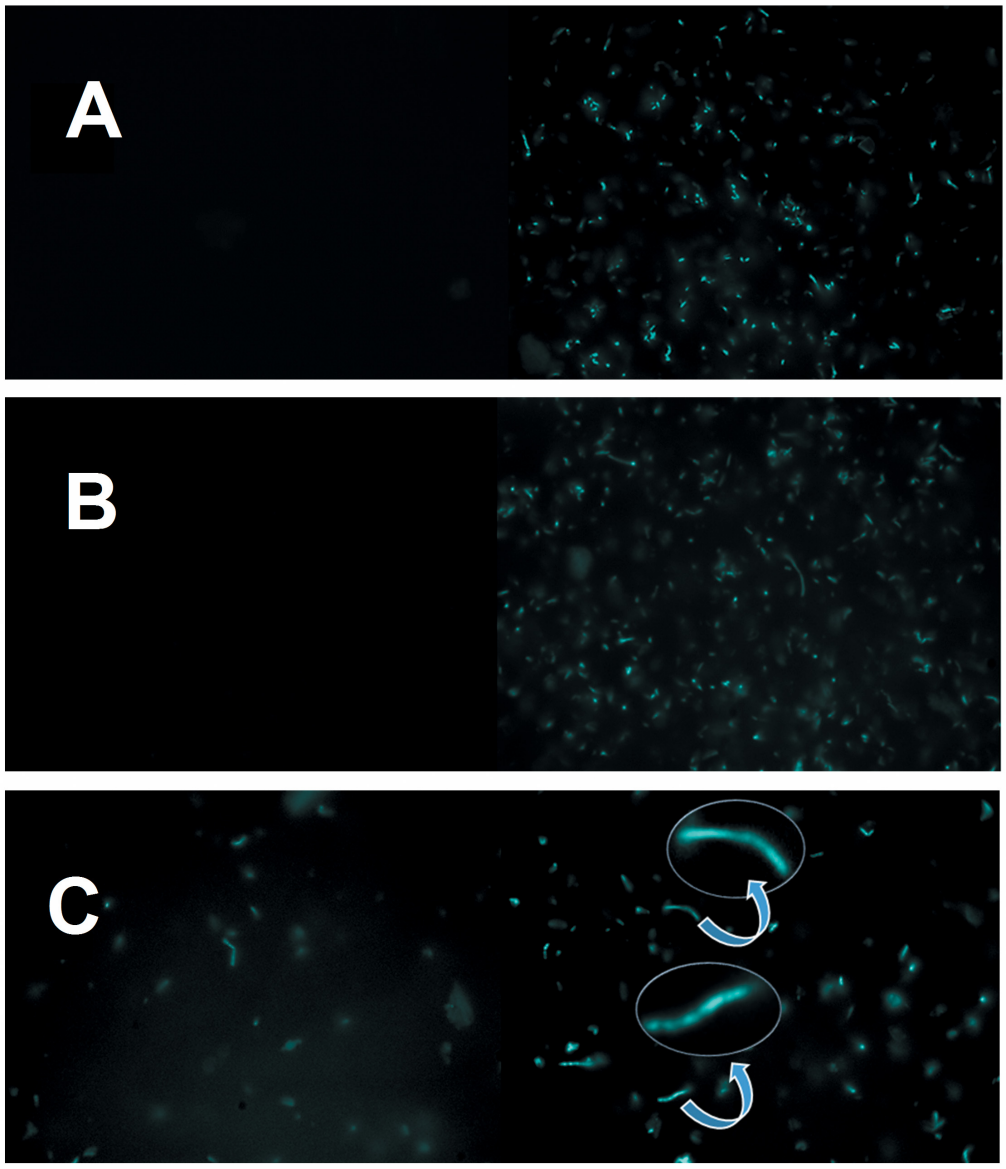
Formation of Cygb amyloid structure after challenge by peroxides. A), B) and C) show, respectively the epifluorescence images of Cygb control, control plus GSH and challenged by hydrogen peroxide obtained immediately (left panels) 24 h (right panels) after incubation and staining by thioflavine-T. For the low-vacuum SEM experiments, it was used 7 μmol.L^-1^ cygb solution with 70 μmol.L^-1^ peroxide solutions. For the epifluorescence experiments 70 μmol.L^-1^ protein solution was incubated for 1 h with 700 μmol.L^-1^ peroxide solution in the presence of thioflavin-T. For FTIR measurements, 7 μmol.L^-1^ protein solution was incubated with 70 μmol.L^-1^ peroxide solutions for 1 h. The results are representative of three independent experiments.

To corroborate the formation of Cygb amyloid fibrils promoted by oxidative processes of the protein, samples of native, GSH- and t-BuOOH-treated Cygb were dyed with the fluorescent dye thioflavin-T (Th-T) and analyzed using epifluorescence microscopy (Fig 10A, 10B and 10C). The fluorescent dye Th-T exhibits an increase of several orders of magnitude upon fibril binding and is considered to be an efficient and sensitive reporter of the formation of amyloid structures both in vivo and in vitro. The intense increase of Th-T fluorescence upon binding to amyloid fibrils results from the binding to fibrils that imobillizes a subgroup of Th-T conformers in grooves produced by ladders of protein side chains, preferentially in those of aromatic amino acid residues [73–77]. In Fig 10A, we observe that fibrils are absent at first and present after 24 h of incubation. Similar results were obtained in the presence of GSH (Fig 10B). However, Cygb incubated with hydrogen peroxide presented fibrilles immediatelly after addition of hydrogen peroxide and large fibrilles were detected after 24 h of incubation (Fig 10C). The inset of Fig 10C shows a zoom-in of two fibrilles. The capacity to form amyloid fibrilles has been previously reported for myoglobin induced by segments of unfolded peptides [78]On the other hand, literature data report protective role of neuroglobin against the formation of amyloid structures in cells [79]. The results obtained for cygb in vitro shows that this globin has potential to form amyloid structures but further studies are necessary to prove the formation of cygb amyloid structures in cells.

**Fig 11:**
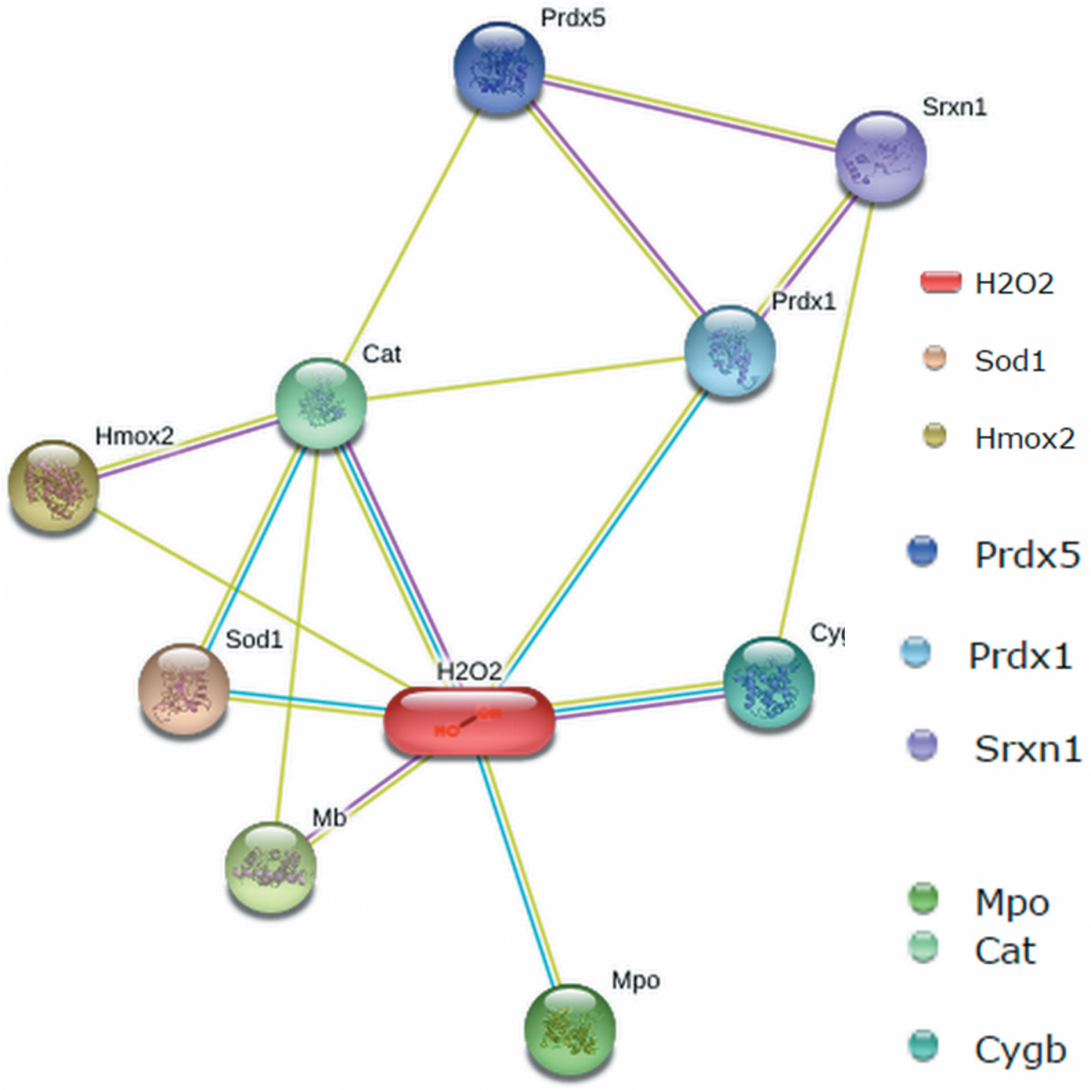
Interatoma of rat Cygb with hydrogen peroxide. The network shows, in each node, a protein predicted to have functional links with Cygb and hydrogen peroxide. Inside the figure the abbreviations are SOD1 (superoxide dismutase [Cu-Zn]), Hmox2 (heme oxygenase 2 [HO-2]), Mb (myoglobin), Mpo (myeloperoxidase), cat (catalase), Cygb (cytoglobin), Prdx1 (peroxyredoxin-1), Prdx5 (peroxyredoxin-5) and Srxn1 (Ab2-390). In the figure light green, cyan and magenta lines correspond, respectively, to textmining, databases and experiments supporting the relationship among the proteins and hydrogen peroxide.

Considering the capacity of Cygb for reacting with peroxides and forming amyloid fibrils, we performed an interatoma of Cygb with hydrogen peroxide (Fig 11) to search for evidence for a functional association between Cygb and hydrogen peroxide. Interactoma with hydrogen peroxide shows a correlation between Cygb and the cell antioxidant apparatus dependent on GSH consumption. Fig 11 shows the interatoma of Cygb with hydrogen peroxide. The network shows, in each node, a protein predicted to have functional links with Cygb and hydrogen peroxide. In the network displayed in Fig 11, the different colors correspond to each type of evidence.

The functional relationship of Cygb with hydrogen peroxide and organic peroxide (linoleic acid-derivative) was evidenced by experiments [25], [26], databases and text mining. Cygb was previously experimentally correlated with hydrogen peroxide and text mining revealed a possible correlation with Srxn1 that in turn is correlated with Prxn1 and Prxn5. Overexpression of Cygb was detected in stellate cells challenged by thioacetamide, an inducer of hepatic fibrosis associated with GSH depletion. Considering the peroxidase activity of Cygb, the authors of that study attributed the overexpression of the hemeprotein to a protective mechanism that could prevent the fibrosis triggered by oxidative stress. However, the results presented here demonstrate that Cygb challenged by hydrogen peroxide and organic peroxides is converted to fibrils and that this process is prevented by GSH in a concentration-dependent manner. The protective role of Cygb peroxidase activity could be dependent on GSH concentration and could contribute to cell fibrosis in a condition in which GSH is depleted. In Fig 9B, we show that the presence of GSH inhibited the formation of Cygb-amyloid fibrils. Therefore, a possible protective role against a large production of peroxide in cells promoted by Cygb peroxidase activity might occur in conditions in which cells are not yet depleted of GSH. The well-known enzyme involved in the reduction of peroxides, glutathione peroxidase (GPx), and the enzymes involved in the repair of thiol oxidation by peroxides, such as Trx and Prx, all of them consume GSH. Thus, in a condition in which high concentrations of peroxides are present, the increase in Cygb expression could contribute to the elimination of the excess peroxides. However, since the concentration of GSH was declining, the amyloid fibril formation by the Cygb molecules could contribute to the degenerative process. Furthermore, in conditions in which the concentration of organic peroxides is also augmented, the use of these compounds as reducing agents for the recycling of Cygb high-valence species could result in the production of peroxyl radicals that could exacerbate the oxidative damages involved in the formation of amyloid fibrils.

## Conclusion

We have demonstrated that Cygb possesses peroxidase activity against hydrogen peroxide and organic peroxides involving a mechanism similar to that described for myoglobin. The peroxidase mechanism of Cygb involved the formation of a tyrosyl intermediate radical, probably centered at Tyr 59. The elucidation of the Cygb amino acid residue that centers the free radical-intermediated requires site-directed mutagenesis in subsequent investigations. On the other hand, the direct detection of organic peroxyl radicals in the early times of the reaction suggests that organic peroxides can also be used as recycling agents for Cygb high-valence species. However, the generation of free radicals by the peroxidase activity of Cygb leads to oxidative damage of the protein and the formation of fibrillar structure. Therefore, although Cygb possesses peroxidase activity and its overexpression can be a response to the increase of peroxide production, further studies are necessary to determine the role of Cygb in liver fibrosis and in other degenerative diseases associated with the formation of protein fibrils. The results presented here suggest that the protective role of Cygb against an increase in peroxide concentration in cells might be dependent of the reducing status of cells. Thus, in a condition in which the GSH concentration is high, Cygb could promote peroxide reduction without contributing to the formation of amyloid structure. However, given GSH depletion, the overexpression of Cygb could contribute to the formation of amyloid fibrils.
